# Functional Polylactide Blend Films for Controlling Mesenchymal Stem Cell Behaviour

**DOI:** 10.3390/polym12091969

**Published:** 2020-08-30

**Authors:** Yuliya Nashchekina, Pavel Nikonov, Alexey Nashchekin, Natalya Mikhailova

**Affiliations:** 1 Institute of Cytology of the Russian Academy of Sciences, Center of Cell Technologies, 194064 Saint Petersburg, Russia; pashka2316@mail.ru (P.N.); natmik@mail.ru (N.M.); 2 Ioffe Institute, Laboratory “Materials and Structures of Solid State Electronics”, 194021 Saint Petersburg, Russia; nashchekin@mail.ioffe.ru

**Keywords:** poly(lactic acid), polyethylene glycol, polyethylene glycol diamine, blend films, mesenchymal stem cells

## Abstract

Polymer blending is a suitable physical modification method to create novel properties of different polymers. Blending polylactic acid (PLA) and polyethylene glycol (PEG) produces materials with a wide range of properties. This study was the first to investigate the effect of different isomeric forms of PLA and PEG with terminal amino groups to obtain biocompatible films for human mesenchymal stem cell cultivation. It has been shown by scanning electron microscopy that the surface topology changes to the greatest extent when using films obtained on the basis of poly(d,l-lactide) and PEG with high molecular weights (15,000 g/mol). In order to obtain thin films and rapid evaporation of the solvent, PEG is mixed with PLA and does not form a separate phase and is not further washed out during the incubation in water. The presence of PEG with terminal hydroxyl and amino groups in blend films after incubation in water was proven using Fourier transform infrared (FTIR) spectroscopy. Results of fluorescence microscopy demonstrated that blend films formed on PLA and polyethylene glycol diamine (PEG-NH2) are more suitable for cell spreading and focal contact formation compared to cells cultured on the surface of pure PLA films or films made from PLA and PEG.

## 1. Introduction

Polylactide (PLA) is an attractive material for use in tissue engineering owing to its good mechanical properties and processability [1]. At the same time, the surface properties of scaffolds based on PLA are not ideal for cell adhesion and growth [2,3]. The polymer chains lack functional groups, and each lactic acid residue contains a pendant methyl group that gives the surface a significantly hydrophobic nature [4]. It is well-known that hydrophilic surfaces support the adhesion and growth of cells [5]. Numerous approaches have been taken for chemically modifying surfaces of PLA films and scaffolds. The methods used to date include the copolymers synthesis, plasma treatment, entrapment of functional groups on polymers by surface hydrolysis or aminolysis and blending [6,7,8].

Polymer blending is a suitable physical modification method to create novel properties of different polymers [9]. Thermodynamic mixing of two or more polymers allows a material with the desired properties to be obtained, eliminating the need for complex polymer development. Many biologically sourced polymers have been explored on PLA blends for use in biomedical products [10]. Natural materials such as collagen, chitosan, hyaluronic acid, and elastin are currently used in biomedical applications [11,12]. Blending natural polymers with a synthetic polymer PLA significantly improves the properties of the obtained blend materials. New blend materials have also been developed with such novel properties such as shape memory and morphology that are not present in the parent polymers.

Polyethylene glycol (PEG) has been widely applied in various medical applications due to its high biocompatibility, satisfactory safety, hydrophilicity, etc. PEG is frequently used in the production of polymer blends [13]. Blending of PLA and PEG changes the properties of the new materials. It has been demonstrated that the addition of PEG significantly increases the elongation at break and softness of PLA [14]. Jacobsen et al. investigated the properties of plasticized PLA with regards to PEG concentration [15]. The authors observed that the addition of PEG to PLA led to decreases in both tensile strength and the elasticity modulus, along with an increase in the percentage of elongation at break. The addition of 10 wt% PEG resulted in an approximately five-times increase in impact resistance compared to pure PLA. Sheth et al. [16] demonstrated that PLA/PEG blends differ from completely miscible to partially miscible depending on the PEG concentration. Below a 50 wt% PEG concentration, the blends achieved higher elongation and lower modulus values. PEG contents higher than 50 wt% result in changes due to the increasing crystallinity of PEG, consequentially increasing the modulus and decreasing the elongation at break. M. Baiardo demonstrated that PLA/PEG blend materials exhibit brittleness characteristics dependent upon the content and molecular weight of the PEG used [17]. Results showed that the plasticizing efficiency increased as the molecular weight of the PEG decreased. The addition of PEG also reduced the melting temperature and glass transition temperature [18].

Not only do the properties and quantity of the PEG affect the blending of materials, but also the structure of the PLA. As the chiral carbon atom exists in the lactyl repeat unit, PLA has three isomeric forms: Poly(d-lactide) (PDLA), poly(l-lactide) (PLLA), and poly(racemic lactide) (PLDA). The PLLA and PDLA have a crystal structure [19]. The stereoregularity of PLA may similarly affect miscibility with PEG and other plasticizers. The phase condition has important implications for medical application. Kulinski et al. studied the blending of semi-crystalline and amorphous PLA with PEG. They demonstrated that the incorporation of PEG within an amorphous plasticized PLA results in considerable deformation, while a semi-crystalline PLA exhibits non-uniform plasticization of the amorphous phase and less ability for plastic deformation [20].

Previously, we demonstrated that, when blending polymers based on amorphous PLA and PEG, the latter remains in the material and affects the scaffold properties [21]. It should also be noted that the presence of PEG in a blend polymer not only affects the physical and mechanical properties, but also the surface properties. The terminal hydroxyl groups of PEG can increase the hydrophilicity of the blend polymer when compared to pure PLA. Increasing the hydrophilicity of the polymer surface can significantly improve the biocompatibility of the material for culturing cells. In our work, we first proposed to improve the properties of PLA using not only PEG with terminal hydroxyl groups, but also PEG with amino groups (PEG-NH2). Both hydroxyl and amino groups increase the hydrophilicity of the surface and, thus, the biocompatibility of the scaffold. The purpose of this study is to obtain films based on a blend of PLA/PEG, as well as PLA/PEG-NH2. It is not only important to study the effect of PEG on blending films, but also the influence of various isomeric forms of PLA on the phase separation of polymers and the properties of blending films. In the course of the work, the properties of the obtained materials will be studied, as well as their prospect for tissue engineering and regenerative medicine.

## 2. Materials and Methods 

### 2.1. Materials

PLLA (IV = 4.9 dL/g) and PDLA (IV = 3.8 dL/g) were purchased from Corbion (Gorinchem, The Netherlands). PEG with molecular weight *M*_n_ = 6000 g/mol and *M*_n_ = 15,000 g/mol were purchased from Sigma-Aldrich (St. Louis, MO, USA) and were used as received. PEG-NH2 with molecular weight *M*_n_ = 6000 g/mol and *M*_n_ = 10,000 g/mol were purchased from Sigma-Aldrich (St. Louis, MO, USA) as well. 

### 2.2. Film Formation

Polymer films were prepared using solvent cast techniques. The PLLA or PDLA concentration in the solution was 0.02 g/mL. Polymer films were prepared by mixing PLLA or PDLA and PEG (70/30, *w*/*w*) in chloroform (Reactiv, Saint-Petersburg, Russia). The 50 µL solution was then cast on a coverslip with a diameter of 11 mm, followed by air drying for 24 h and vacuum drying for the next 72 h. The dispersed PEG phase was eliminated by dissolution in distilled water at 25 °C for 24 h (0.1 g film in 100 mL water). The thickness of the PLA films was determined to be 5 μm. The films for cell cultivation were sterilized using a 200 ppm ozone gas for 120 min at 80% relative humidity. 

### 2.3. FTIR

The surface molecular structure of PLA/PEG blend samples was analyzed using a Fourier transform infrared (FTIR) spectrometer IRPrestige-21 (Shimadzu, Tokyo, Japan), in transmission mode, in the 4000–600 cm^−1^ range, and with spectral resolution 2 cm^−1^.

### 2.4. Scanning Electron Microscopy

The structure of the PLA/PEG blend samples surface was evaluated using a JSM-7001F (Jeol, Tokyo, Japan) scanning electron microscope (SEM).

### 2.5. Water Contact Angle

The static contact angles of water were measured at room temperature on a contact angle, using the sessile drop method. The essence of the measurement was as follows. A film sample was glued onto a glass slide. Then, 15 μL of distilled water was applied to the test sample with a special automatic dispenser. Using the device’s camera, the droplet shape on the test sample was evaluated and the contact angle calculated.

### 2.6. MSC Cultivation 

The non-transformed fetal mesenchymal stromal cells (FetMSC) cell line obtained from the Vertebrate Cell Culture Collection (Institute of Cytology RAS, Saint Petersburg, Russia) was used as a human MSC line. This cell line was derived from stromal cells of a 5–6 week fetal bone marrow and was characterized according to the minimal criteria proposed by the International Society of Cell Therapy for defining human MSCs. The cells adhered well to plastic culture dishes; expressed surface markers, such as CD73, CD90, and CD105; did not express CD34 and HLA-DR markers; and had the ability to differentiate in vitro in the osteogenic and adipogenic directions. Karyotypic analysis carried out at passages 12–14 showed normal karyotype 46, XY. 

### 2.7. Fluorescence Staining of FetMSCs 

FetMSCs were fluorescence stained in order to study the effects PEG and PEG-NH2 modification on FetMSC adhesion, spreading, and the presence of focal contacts. In this experiment, pure glass was used as a control.

FetMSCs were seeded and cultivated on the blend films at 37 °C in a CO_2_ incubator for either 2 h. After the cultivation period, the medium was removed and the adherent cells were washed with phosphate buffered saline (PBS), fixed with a 4% formaldehyde solution (Sigma-Aldrich, St. Louis, MO, USA) for 10 min, and then washed three times with PBS. Next, a detergent solution consisting of 0.1% Triton X-100 (Sigma-Aldrich, St. Louis, MO, USA) was added to the cells for 15 min and then washed off with PBS. Samples were then stained with rhodamine phalloidin (Thermo Fisher Scientific, Carlsbad, CA, USA) for 15 min, in order to stain the cytoskeleton, and then washed with PBS. Finally, the FetMSCs were treated with a mounting-medium containing DAPI (4′,6-diamidino-2-phenylindole) (ab104139; Abcam, Cambridge, MA, USA). The actin cytoskeleton organization was then observed using a confocal microscope (Olympus FV3000, Tokyo, Japan).

To study the presence of focal contacts by FetMSCs cultivated on the blend films, the cells were incubated with anti-vinculin antibodies. Following 5 h or 1 day of cultivation, the culture medium was removed and the cells on modified films were washed with PBS. The cells were then fixed with a formalin solution for 10 min and then washed three times with a 0.1% Tween PBS solution. Next, the cells were treated with 0.1% Triton X-100 for 15 min and then washed again three times with a 0.1% Tween PBS solution. After the final washing process, anti-vinculin rabbit antibodies (ab129002; Abcam, Cambridge, MA, USA) were added to the cells for a 24 h period. Following this incubation, the polymer films were carefully washed with PBS and then goat anti-rabbit IgG (H & L chain) antibodies (ab205718; Abcam, Cambridge, MA, USA) were added for 45 min. Next, the cells were washed with PBS containing 0.1% Tween and then the cells were treated with a mounting-medium containing DAPI. Then, using a confocal microscope (Olympus FV3000, Tokyo, Japan), we observed the cells for the presence of focal contacts.

### 2.8. Cell Area Spreading and Focal Contacts Counts

To study the effects of the blend films on cellular spreading, the number of focal contacts was cultured for a period of either 2, 5 h, or 1 day. For this purpose, five different pictures of fields on each of the polymer surface were taken using a using a confocal microscope. The ImageJ program was used to count the cell area spreading and the number of focal contacts [22].

### 2.9. Statistical Analysis

All experiments were performed in 3–5 replicates. The t-test was performed by using the Microsoft Excel Software to analyze the statistically significant differences between specific samples. Samples were considered to be statistically important with the *p* < 0.05.

## 3. Results and Discussion

**Figure 1:**
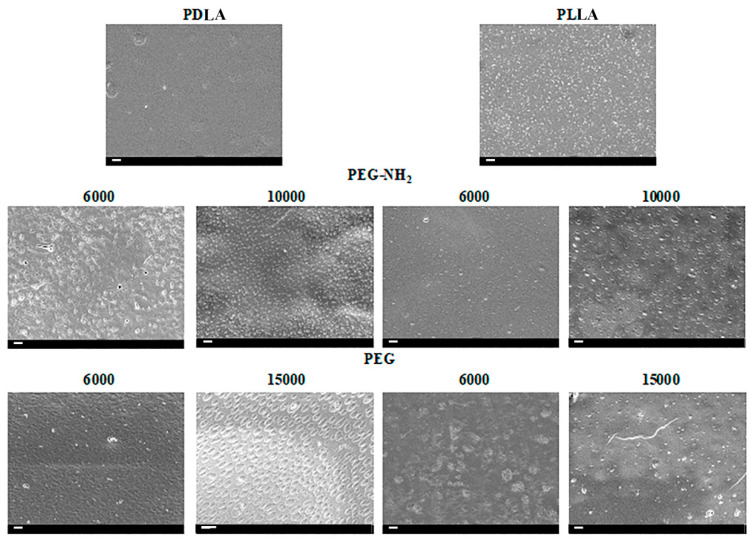
Scanning electron microscopy (SEM) images of pure polylactide (PLA) with different isomeric forms—poly(d,l-lactide) (PDLA) and poly(l,l-lactide) PLLA; the blend polylactide/polyethylene PLA/PEG or polylactide/polyethylene glycol diamine (PLA/PEG-NH2) with different molecular weights of PEG (6000 or 15,000 g/mol) and PEG-NH2 (6000 or 10,000 g/mol). Scale bar 1 µm.

Polymer blending is a method that allows new materials to be formed with improved characteristics [9]. As a result of blending PLA and PEG, two phases are formed [14]. After evaporation of the solvent, the resulting composite is incubated in water, which the PEG phase becomes partially dissolved in. It is known that the formation of a two-phase system depends on many factors, including the molecular weight of PLA and PEG, the properties of the solvent, the mixing speed, and the evaporation time of the solvent [9,21]. The aim of this study was to obtain blend films with a high content of hydroxyl and amino groups, the source of which were PEG and PEG-NH2, respectively. We assumed that different isomeric forms of PLA would affect the properties of the blend films. For a uniform distribution of PEG and PEG-NH2 in the blend films, the solution was thoroughly mixed and covered by a thin glass film. As a result of the rapid evaporation of chloroform, complete separation of the PLA and PEG phases did not occur. Figure 1 shows the SEM micrographs of the surface PLA/PEG blend films. 

**Figure 2:**
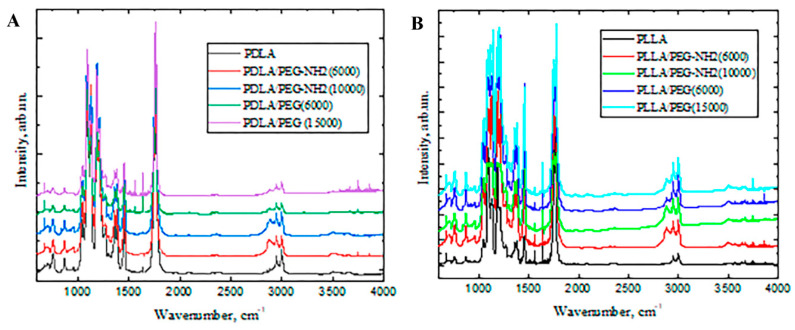
Fourier transform infrared (FTIR) spectra of the pure PLA with different isomeric forms—PDLA (**A**) and PLLA (**B**); the blend PLA/PEG or PLA/PEG-NH2 with different molecular weights of PEG (6000 or 15,000 g/mol) and PEG-NH2 (6000 or 10,000 g/mol).

The SEM results demonstrate an absence of pores with only shallow wells present in samples containing higher molecular weight PEG: PEG-NH2 with a molecular weight of 10,000 and PEG with a molecular weight of 15,000. The largest diameter holes were observed in samples obtained from mixing PDLA and PEG (15,000). The diameter of the holes in these samples reached 0.5–1 µm. The holes are evenly distributed across the surface of the film. The surfaces of films produced from a mixture of PEG (15,000) and PLA in another isomeric form, PLLA, also contained holes, however the diameter of those did not exceed 100 nm and the number of holes was significantly lower than on blend films produced from PLA in the isomeric form of PDLA. PEG-NH2 with terminal amino groups and high molecular weight (10,000) also contributed to the formation of shallow wells when mixed with PLA of different isomeric forms. As shown in Figure 1, the diameter of these holes reaches 0.5 µm, while the number of holes on the surface of films from different isomeric forms of PLA differs significantly. The number of holes on the surface of blend films from a mixture of PDLA/PEG-NH2 (10,000) is several times greater than the number of holes on the surface of films obtained from a blending of PLLA/PEG-NH2 (10,000). Using PEG with a lower molecular weight does not allow one to obtain films with holes on the surface. Moreover, both PEG and PEG-NH2 with molecular weights of 6000 do not form separate phases that can be dissolved in water. However, the SEM results show that the surface of the sample obtained from a blending of PDLA/PEG-NH2 (6000) has a relief topology, which may be the cause of the phase separation of PDLA and PEG-NH2 (6000). A similar result was observed on the surface of the sample obtained on the basis of a blending of PLL and PEG (6000). The surface of this sample also had irregularities that may be due to the phase separation of PLLA and PEG (6000). Thus, it can be concluded that, under these conditions and rapid evaporation of chloroform, phase separation only occurs when blending PLA in the isomeric form of PDLA and using PEG with a high molecular weight. It should also be noted that, when using PEG-NH2 with terminal amino groups, the diameter of the holes formed on the surface of blend films is several times smaller than the diameter of the holes formed on the surface of films obtained from a blending of PLA and PEG. One can find works on the formation of porous films based on PLLA and PEG with different molecular weights [23]. With a prolonged evaporation of the solvent, the two phases become completely separated and porous structures are formed [24,25]. Despite the fact that there are works on the influence of different molecular weights of PEG on pore size, data on the influence of the isomeric form of PLA used in the composition of the PLA/PEG blend are not available in the literature. We demonstrated that, not only PEG, but also the structure of the PLA affects the surface properties of the films produced from PLA/PEG blends. In our previous studies, using photometry and NMR, we showed that even after long-term incubation of blend films in water the PEG remains in the films and affects the film properties [21]. Similar results have been obtained by other researchers [14]. In this work, to confirm the presence of PEG and PEG-NH2 in the blend films after incubation in water, FTIR spectra of pristine PLA, PLA/PEG blend, and PLA/PEG-NH2 were evaluated, as shown in Figure 2. 

PLA shows characteristic stretching frequencies for C=O, –CH3 asymmetric, –CH3 symmetric, and C–O at 1746, 2995, 2946, and 1080 cm^−1^, respectively. Blending frequencies for –CH3 asymmetric and –CH3 symmetric have been identified at 1452 and 1361 cm^−1^, respectively. The spectra obtained by us coincides with the previously obtained data for PLA by other researchers [25].

Peaks were observed at 2878 and 3446 cm^−1^ for PEG, corresponding to the C-H and terminal OH group, respectively. It should also be noted that the intensity of the 2878 cm^−1^ peak, characteristic of C–H in mixed films based on PLA and PEG with terminal amino groups, is higher than the signal intensity for films based on a blending of PLA and PEG. This may be evidence of a larger number of C–H groups in the samples based on PLA and PEG with terminal amino groups.

According to the literature data, the main bands corresponding to the amino groups are 3500–3300 cm^−1^ for stretching and 1650–1580 cm^−1^ for deformation [26]. The presence of signals in the specified ranges on the presented spectra indicates the presence of amino groups in PLA/PEG-NH2 blend films after incubation in water for 1 day.

**Figure 3:**
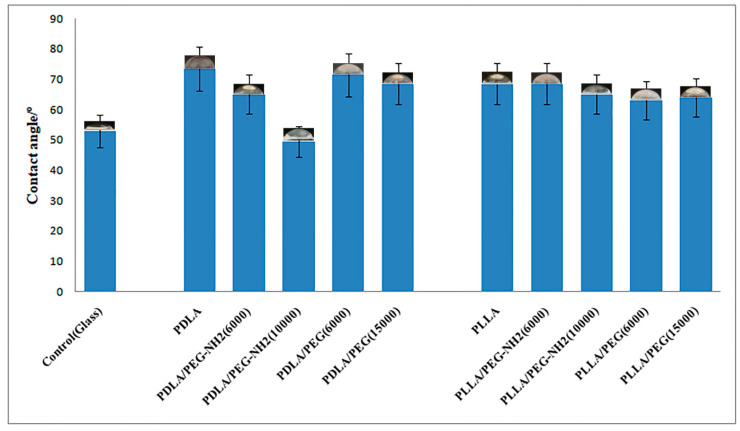
Contact angles of the pure PLA with different isomeric forms—PDLA and PLLA; the blend PLA/PEG or PLA/PEG-NH2 with different molecular weights of PEG (6000 or 15,000 g/mol) and PEG-NH2 (6000 or 10,000 g/mol).

As mentioned above, some of the main disadvantages of PLA are its hydrophobicity and lack of functional groups. FTIR spectroscopy demonstrated that blending PLA with PEG containing OH-terminal groups and PEG-NH2 containing amino-terminal groups improve film properties. An important property of the surface of polymer films is their hydrophilicity or contact wetting angle. A water contact angle formed in the range of 40–70° on a polymeric surface is known to influence cell attachment, since chemical surface interactions are a key factor during the bio-adhesion process [9]. Polymers with contact angles in this range were thus selected for this analysis, and their hydrophilicity was slightly modified by blending. From the results shown in Figure 3, we can discern that the most hydrophobic sample was PDLA with a contact angle of 73°. When blending PDLA with the more hydrophilic PEG-NH2 (10,000), the surface wettability improved 22% to a value of 51°. The addition of PEG or PEG-NH2 in the remaining samples did not lead to a statistically significant reduction in the contact angle. This may be due to the fact that the terminal amino groups in this method of producing blend films do not provide a maximum number of functional groups on the surface. This method of forming films should be optimized in future studies.

Reviewing the data in the currently available literature reveals that the surface chemistry and charge character are also very important factors in terms of cellular adhesion. It seems that carboxylic acid groups grafted on the polymer surface have negative effects on cell spreading, and growth, which is consistent with previous suggestions by others researchers [8,27]. To evaluate the biocompatibility of films formed by blending PLA and PEG or PEG-NH2 on modified films, human FetMSC cells obtained from the bone marrow of a healthy donor were cultured for one day. 

**Figure 4:**
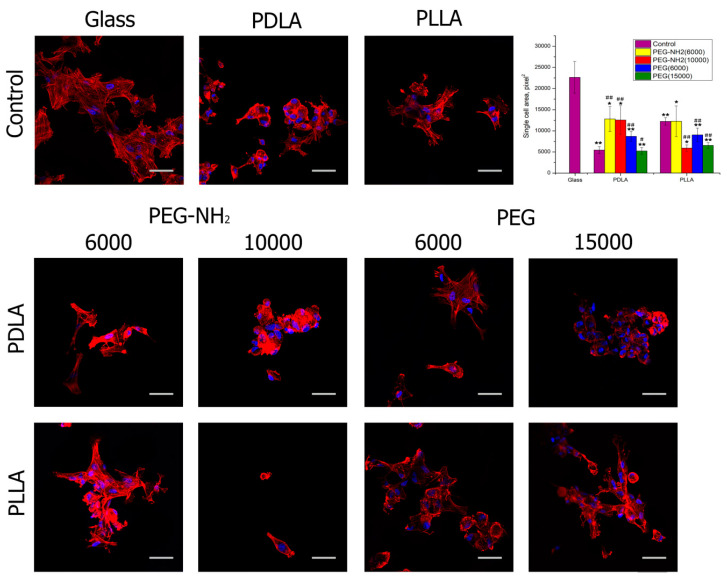
Fluorescence microscopy of the fetal mesenchymal stromal cells (FetMSC)s after 2 h of cultivation on the pure glass and on the pure films from PLA with different isomeric forms—PDLA and PLLA (control); the blend PLA/PEG or PLA/PEG-NH2 with different molecular weights of PEG (6000 or 15,000 g/mol) and PEG-NH2 (6000 or 10,000 g/mol). N = 5: *—*p* < 0.01, **—*p* < 0.05 for the same concentration data, #—*p* < 0.01, ##—*p* < 0.05 compared with the unmodified PCL. Staining—rhodamine-phalloidin (red), 4′,6-diamidino-2-phenylindole (DAPI) (blue). Scale bar 50 µm.

After 2 h of cultivation and staining of actin filaments with rhodamine-phalloidin, the degree of spreading was assessed (Figure 4). This figure shows that, after 2 h of cultivation, almost all samples contained cells attached and well spread out. In comparison to untreated films, the best results were observed when blending PDLA and PEG-NH2 with a molecular weight of 6000 or 10,000. Moreover, the terminal amino groups of PEG have a better effect on the degree of cell spreading than the terminal hydroxyl groups. Earlier in our research, as well as in the work of others, it has been shown that the positive charge of amino groups on the surface of polymer films increases cell adhesion and proliferation [8,28]. Using films based on a blend of PLLA and PEG with terminal hydroxyl and amino groups, the positive effects of PEG on cell spreading were not observed after 2 h of cultivation. It should be noted that, in these films, the presence of PEG similarly did not have a positive effect on the wetting angle. This once again confirms our assumption that, when using an isomeric PLLA form and “rapid” evaporation of the solvent, there is no separation of the PEG phases; the terminal hydroxyl and amino groups of PEG remain in the film volume and do not affect the surface properties of the film.

**Figure 5:**
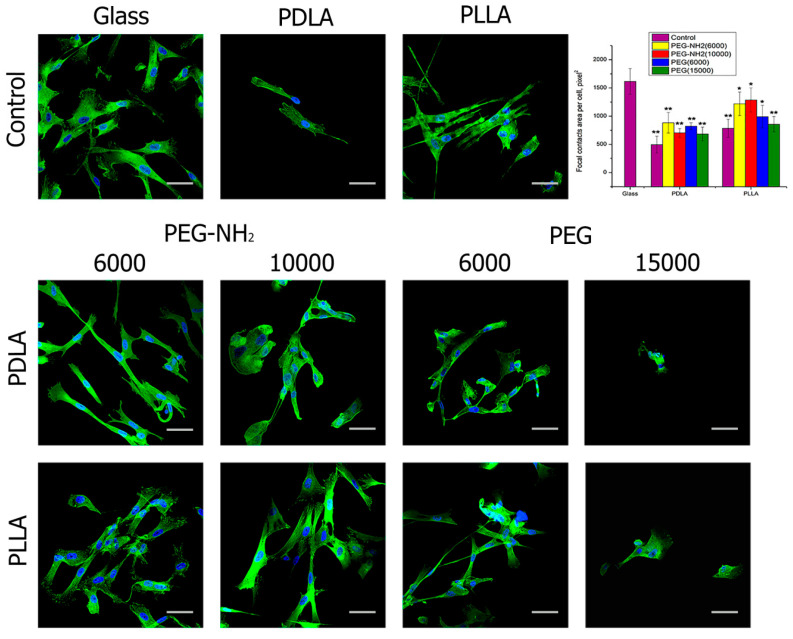
Presence of focal contacts of the MSC cells after one day of cultivation on the pure glass and on the pure films from PLA with different isomeric forms—PDLA and PLLA (control); the blend PLA/PEG or PLA/PEG-NH2 with different molecular weights of PEG (6000 or 15,000 g/mol) and PEG-NH2 (6000 or 10,000 g/mol). (N = 5: *—*p* < 0.01, **—*p* < 0.05 for the same concentration data). Staining—vinculin (green), DAPI (blue). Scale bar 50 µm.

After one day of cultivation, a positive effect in terms of the number of focal contacts was observed when blending PEG-NH2 with either isotropic form of PLA, PDLA, and PLLA (Figure 5). PEG with hydroxyl functional groups did not have a significantly positive effect on the formation of adhesion contacts in cells. Despite the fact that antibodies to vinculin have been used for a long time to assess the biocompatibility of synthetic materials in relation to cells, the mechanism of influence of functional groups of both hydroxyl and amino groups has yet to be studied.

**Figure 6:**
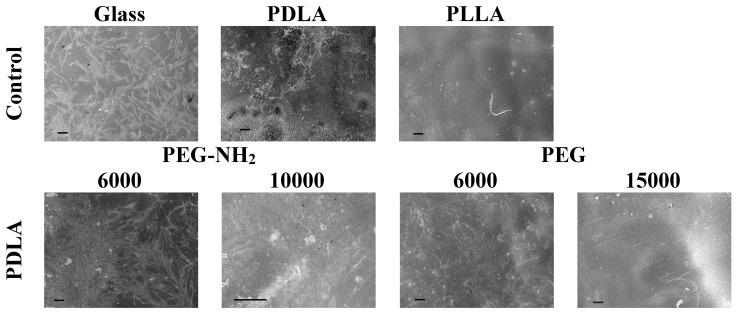

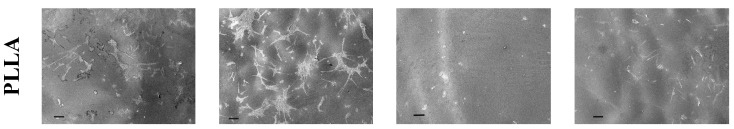
SEM images of the MSC cells after one day of cultivation on the pure glass and on the pure films from PLA with different isomeric forms—PDLA and PLLA (control); the blend PLA/PEG or PLA/PEG-NH2 with different molecular weights of PEG (6000 or 15,000 g/mol) and PEG-NH2 (6000 or 10,000 g/mol). Scale bar 100 µm.

After one day of cultivation, cell morphology was studied using SEM (Figure 6). The obtained results confirmed the data from the fluorescence microscopy studies. The largest number of cells comparable to the control (glass) were observed on polymer films based on blends of PLA and PEG-NH2. The cells were well spread out and a monolayer was almost formed on the surface of the film obtained by blending PDLA and PEG-NH2 (6000). On the surfaces of the films formed by blending PLA and PEG, the number of attached and spread cells were insignificant.

## 4. Conclusions

In the course of this work, for the first time, we formed and studied films using PLLA or PDLA and PEG or PEG-NH2 with terminal hydroxyl or amino groups. When forming thin films, the solvent is rapidly evaporated and PEG does not have time to form a separate phase, thus it blends to the PLA macromolecule. After film incubation in water, PEG remains in the blending. We have demonstrated by FTIR spectroscopy that, during this film formation method, the terminal hydroxyl and amino groups of PEG remain in the blend films and affect their surface and biological properties. It has been shown by SEM that the use of PEG with both terminal hydroxyl and amino groups, however with a high molecular weight (15,000 and 10,000, respectively), makes it possible to obtain blend films with a more developed topology compared to films obtained from PLA and PEG with a lower molecular weight (6000). Moreover, when blending the isomeric form PDLA and PEG, a greater degree of phase separation was observed between the polymers when compared to PLLA and PEG. In films formed by blending PDLA and PEG, an increase in the hydrophilicity of the surface was observed in comparison with films formed by blending PLLA and PEG. When studying the biocompatibility of the obtained blend films, the highest degree of flattening and the greatest number of focal contacts was observed in cells cultivated on blend films obtained by blending PLA and PEG-NH2 with terminal amino groups. This confirms the previously obtained results regarding the attractiveness of amino groups on the surface of PLA films for the cultivation of mesenchymal stem cells.

## References

[1] Poh P.S.P., Chhaya M.P., Wunner F.M., De-Juan-Pardo E.M., Schilling A.F., Schantz J.T., van Griensven M., Hutmacher D.W. (2016). Polylactides in additive biomanufacturing. Adv. Drug Deliv. Rev..

[2] Wang S., Cui W. (2005). Bulk and surface modifications of polylactide. J. Anal. Bioanal. Chem..

[3] Shved Y.A., Kukhareva L.B., Zorin I.M., Bilibin A.Y., Blinova M.I., Pinaev G.P. (2007). Interaction of cultured skin cells with the polylactide matrix coved with different collagen structural isoforms. Cell Tiss. Biol..

[4] De Bartolo L., Morelli S., Bader A., Drioli E. (2002). Evaluation of cell behaviour related to physico-chemical properties of polymeric membranes to be used in bioartificial organs. Biomaterials.

[5] Nashchekina Y.A., Alexandrova S.A., Nikonov P.O., Ivankova E.I., Yudin V.E., Blinova M.I., Mikhailova N.A. (2019). Study of the Osteoindictive Properties of Protein-Modified Polylactide Scaffolds. Bull. Exp. Biol. Med..

[6] Chu P.K., Chen J.Y., Wang L.P., Huang N. (2002). Plasma-surface modification of biomaterials. Mater. Sci. Eng. R.

[7] Korzhikov-Vlakh V., Averianov I., Sinitsyna E., Nashchekina Y., Polyakov D., Guryanov I., Lavrentieva A., Raddatz L., Korzhikova-Vlakh E., Scheper T. (2018). Novel Pathway for Efficient Covalent Modification of Polyester Materials of Different Design to Prepare Biomimetic Surfaces. Polymers.

[8] Nashchekina Y., Chabina A., Nashchekin A., Mikhailova N. (2020). 2020 Polycaprolactone Films Modified by L-Arginine for Mesenchymal Stem Cell Cultivation. Polymers.

[9] Saini P., Arora M., RaviKumar M.N.V. (2016). Poly(lactic acid) blends in biomedical applications. Adv. Drug Del. Rev..

[10] Pawar R.P., Tekale S.U., Shisodia S.U., Totre J.T., Domb A.J. (2014). Biomedical applications of poly(lactic acid). Recent Pat. Regen. Med..

[11] Catoira M.C., Fusaro L., Di Francesco D., Ramella M., Boccafoschi F. (2019). Overview of natural hydrogels for regenerative medicine applications. J. Mater. Sci. Mater. Med..

[12] Nashchekina Y.A., Yudintceva N.M., Nikonov P.O., Ivanova E.A., Smagina L.V., Voronkina I.V. (2017). Effect of Concentration of Collagen Gel on Functional Activity of Bone Marrow Mesenchymal Stromal Cell. Bull. Exp. Biol. Med..

[13] Garric X., Molès J.P., Garreau H., Guilhou J.J., Vert M. (2005). Human skin cell cultures onto PLA50 (PDLLA) bioresorbable polymers: Influence of chemical and morphological surface modifications. J. Biom. Mater. Res. A.

[14] Hu Y., Hu Y.S., Topolkaraev V., Hiltner A., Baer E. (2003). Aging of poly(lactide)/poly(ethylene glycol) blends. Polymer.

[15] Jacobsen S., Fritz H.G. (1999). Plasticizing polylactide—The effect of different plasticizers on the mechanical properties. Polym. Eng. Sci..

[16] Sheth M., Kumar R.A., Dave V., Gross R.A., McCarthy S.P. (1997). Biodegradable polymer blends of poly (lactic acid) and poly (ethylene glycol). J. Appl. Polym. Sci..

[17] Baiardo M., Frisoni G., Scandola M., Rimelen M., Lips D., Ruffieux K., Wintermantal E.J. (2003). Thermal and mechanical properties of plasticized poly (l-lactic acid). Appl. Polym. Sci..

[18] Ljungberg N., Wesslen B. (2002). Plasticization of poly(lactic acid) with oligomeric malonate esteramides: Dynamic mechanical and thermal film properties. J. Appl. Polym. Sci..

[19] Jun S., Yan-Long L., Sheng X., Xin-Chao B., Jing-Ru S., Gao L., Chen X.S., Hou H.Q. (2015). The stereocomplex formation and phase separation of PLLA/PDLA blends with different optical purities and molecular weights. Chin. J. Polym. Sci..

[20] Kulinski Z., Piorkowska E. (2005). Crystallization, Structure and Properties of Plasticized Poly(l-Lactide). Polymer.

[21] Nashchekina Y., Samusenko I., Zorin I., Kukhareva L., Bilibin A., Blinova M. (2019). Poly(d,l-lactide)/PEG blend films for keratinocyte cultivation and skin reconstruction. Biomed. Mater..

[22] Horzum U., Ozdil B., Pesen-Okvur D. (2014). Step-by-step quantitative analysis of focal adhesions. MethodsX.

[23] Li F.-J., Liang J.-Z., Zhang S.-D., Zhu B. (2015). Tensile Properties of Polylactide/Poly(ethylene glycol) Blends. J. Polym. Environ..

[24] Nakane K., Hata Y., Morita K., Ogihara T., Ogata N. (2004). Porous poly(l-lactic acid)/poly(ethylene glycol) blend films. J. Appl. Polym. Sci..

[25] Tsuji H., Smith R., BonfieldWand Ikada Y. (2000). Porous biodegradable polyesters. Preparation of porous poly(l-lactide) films by extraction of poly(ethylene oxide) from their blends. J. Appl. Polym. Sci..

[26] Woei Chieng B., Azowa Ibrahim N., Md Zin Wan Yunus W., Zobir Hussein M. (2014). Poly(lactic acid)/Poly(ethylene glycol) Polymer Nanocomposites: Effects of Graphene Nanoplatelets. Polymers.

[27] Cao B., Peng Y., Liu X., Ding J. (2017). Effects of Functional Groups of Materials on Nonspecific Adhesion and Chondrogenic Induction of Mesenchymal Stem Cells on Free and Micropatterned Surfaces. ACS Appl. Mater. Interfaces.

[28] Jeznach O., Kolbuk D., Sajkiewicz P. (2019). Aminolysis of various aliphatic polyesters in a form of nanofibers and films. Polymers.

